# Hyperactive Catatonia in an Adolescent With Prader-Willi Syndrome

**DOI:** 10.7759/cureus.82304

**Published:** 2025-04-15

**Authors:** Rame Alharbi, Saeed S Shaaban, Eric MacMaster

**Affiliations:** 1 Department of Psychiatry, State University of New York Upstate Medical University, Syracuse, USA

**Keywords:** agitation, behavior change, catatonia, child and adolescent psychiatry, hyperactive catatonia, pediatric genetics, prader-willi, prader-willi syndrome

## Abstract

Catatonia, a neuropsychiatric syndrome, has been increasingly recognized as a possible complication of Prader-Willi syndrome (PWS). There is limited research surrounding catatonia and its sequelae in PWS. Given the scarcity and severity of catatonia in pediatric age, there is a need to expand on the available literature. We present a case of hyperactive catatonia in an adolescent with PWS. After obtaining a thorough history, we followed her progression from motor symptoms to developing psychotic features. Although her presentation required multiple doses of lorazepam, it was shown to be consistently effective in treating her catatonia during her hospital stay.

## Introduction

Prader-Willi syndrome (PWS), a rare genetic syndrome, is characterized by errors of genomic imprinting resulting from the absence of paternally inherited genes in the 15q11-q13 region. The typical presentation is attributed to either paternal deletion or maternal uniparental disomy, resulting in both chromosome 15s being inherited from the mother [1]. PWS is characterized by various physical, behavioral, and psychiatric features, including profound hypotonia, hyperphagia, eating unusual food items, self-injurious behavior, and cognitive deficits [2]. An association has been noted between PWS, particularly in individuals with maternal uniparental disomy, and neuropsychiatric conditions in which they were at a higher risk of developing psychosis and catatonia [3].

Catatonia, a neuropsychiatric syndrome characterized by motor, behavioral, and autonomic abnormalities, is increasingly recognized in pediatric populations with PWS [4,5]. Its symptoms may include mutism, stupor, rigidity, posturing, and waxy flexibility [6]. Catatonia can present across a wide clinical spectrum, from marked unresponsiveness and immobility (e.g., stupor, mutism, and waxy flexibility) to excessive, often purposeless or bizarre, motor activity such as stereotypy, agitation, echolalia, and echopraxia [7]. This hyperactive or agitated subtype, often underrecognized, is particularly relevant in pediatric patients, whose presentation may fluctuate between hypoactive and hyperactive features [3,7]. It is commonly associated with psychiatric illnesses, including schizophrenia, bipolar disorder, and autism, and can also arise from various medical conditions such as neoplasms, autoimmune diseases, medications, and metabolic disturbances [6]. Dhossche et al. [3] and Verhoeven and Tuinier [5] explored the link between catatonia, PWS, and the gamma-aminobutyric acid (GABA) system. It has been proposed that a decrease in GABA receptor subunits, resulting from deletions of three subunit genes in the 15q11-q13 region, may predispose individuals with PWS to tantrums, psychosis, and catatonia [1].

Limited publications have been published on the prevalence of PWS and catatonia. One case report published in 1997 [8] described a 17-year-old patient who presented with catatonia and responded to pharmacotherapy alone, while another published in 2015 [9] details a 25-year-old patient who had to be treated with electroconvulsive therapy to show improvement from her catatonic state. The objective of this case report is to detail the course and treatment of a pediatric PWS patient who presented with hyperactive catatonia.

## Case presentation

The patient is a 14-year-old adolescent girl with Prader-Willi and narcolepsy who presented to the pediatric emergency department with her parents for acute behavioral changes for the past nine days. Her presentation was characterized by dyskinetic movements, strange posturing, echolalia, paranoid behavior, and behavioral dysregulation. She had no prior psychiatric history and no issues with abnormal movements or echolalia in the past.

The patient was born as part of a twin pregnancy and first raised concerns for genetic abnormalities at 14 months of age due to growth restriction, microcephaly, and developmental delay compared to her twin sibling. However, genetic testing at that time was inconclusive. At the age of six, she was reevaluated due to persistent failure to thrive, developmental delays, and behavioral issues such as food-seeking and skin picking. Chromosomal analysis at that point confirmed a diagnosis of PWS caused by maternal uniparental disomy (UPD). Socially, she lives with her parents and siblings, who describe her baseline behavior as active within the family and pleasant to be around.

The patient appeared to be her usual self until her parents noticed abnormal movements. She would turn her head to one side and remain locked in that position for extended periods. Erratic neck and eye movements soon followed, and over time, her symptoms worsened. She began adopting awkward sitting positions, such as sitting with her legs raised in the air for hours. Her symptoms would fluctuate, with periods of improvement and relapse, but her repetitive behaviors became more pronounced. She exhibited heightened agitation, paranoia regarding others, accused her sister of being the purple witch, displayed echolalia, and showed facial grimacing asymmetry. The facial drooping, which initially caused her to eat using only one side of her mouth, alarmed her parents, who suspected a stroke. However, the drooping alternated between sides over time.

Before presenting at our facility, she was brought by her parents to a local emergency department due to persistent behavioral irregularities. She exhibited incessant talking, echolalia, and delusional thoughts. Initial laboratory workup, including complete blood count, comprehensive metabolic panel, thyroid function tests, lipase, urinalysis, erythrocyte sedimentation rate, and C-reactive protein, yielded unremarkable results. Toxicology and infectious screenings for Lyme disease, Anaplasma, and Babesia were also normal. A head CT showed no abnormalities. She had not started any new medications but was on somatropin 2.2 mg subcutaneously every night for her PWS as well as pitolisant 35.6 mg orally every morning for her narcolepsy. She was administered two doses of lorazepam totaling 3 mg in the emergency department, which resulted in improvement, and was subsequently referred to our hospital for further evaluation and observation.

Upon admission to our hospital, the patient exhibited fluctuating behaviors, alternating between agitation and a catatonic-like state. The Pediatric team consulted Neurology for further evaluation, which included a neurological examination, lumbar puncture, MRI, laboratory tests, and EEG, all yielding unremarkable results. Notably, prior to the EEG, the patient was administered midazolam 20 mg orally, after which she showed slight clinical improvement. Subsequently, the psychiatry team was involved for additional assessment.

During our initial encounter, the parents reported that the patient had frequent sick contacts over the past month and a recent illness marked by hoarseness and voice changes. However, no other signs of infection were noted. They observed some behavioral improvement following lorazepam administration at the previous hospital. While hospitalized, the patient displayed abnormal, purposeless movements but intermittently engaged playfully, which her parents described as consistent with her baseline behavior. On examination, she presented as a young female adolescent with frequent abnormal, purposeless movements and some forcefulness to her interactions with some staff. Her speech was characterized by echolalia, with reasonable volume, but she would answer questions infrequently. Thought process appeared disorganized, and the patient did not endorse any thoughts of self-harm or harming others. No abnormal or perceptual disturbances were elicited during examination. The patient did not elicit an answer when asked about her mood. Her affect was euthymic, broad, expansive, and inappropriate. Insight and judgment were both limited. A Bush-Francis Catatonia Rating Scale was performed, in which the patient scored 12 points on the screening aspect and a total of 20 points, including the severity score. Based on these findings, she was started on lorazepam at a dose of 2 mg twice daily orally.

The following day, she showed a positive response to treatment, though she appeared sedated. Her parents observed further improvement, with the patient resembling her baseline behavior. Her Bush-Francis score improved to 12, and the lorazepam dose was reduced to 1 mg twice daily. The next day, the patient's mental status had fully returned to baseline as confirmed by her parents. She scored 0 on the Bush-Francis Catatonia Rating Scale, with no psychotic symptoms observed.

Later that day, her parents reported a relapse of erratic behavior around 5 pm, which resolved following her evening dose of lorazepam. In response, an additional midday dose was introduced. By the next day, her Bush-Francis Catatonia Rating Scale score remained 0, with no behavioral or psychotic symptoms observed.

On final evaluation, the patient continued to improve with the addition of the midday dose and, according to her mother, is almost fully recovered. The patient was able to report feeling “fine” and was able to track the conversation between the patient’s mother and the team. The patient also denied any side effects from her medications. Prior to discharge, the patient scored a 3 on the Bush-Francis Catatonia Rating Scale due to holding a position for less than a minute, as well as abnormality in two vitals. Her parents were educated on the importance of follow-up care and informed about plans to gradually wean her off lorazepam as an outpatient. She was discharged that day on lorazepam 1 mg orally in the morning, 0.5 mg orally with lunch, and 1.5 mg orally nightly for a total dose of 3 mg daily. An outpatient appointment was scheduled with neurology and psychiatry for follow-up.

## Discussion

Research on catatonia in pediatric populations is constrained by a significant challenge: the rarity of cases limits opportunities for systematic analysis, which informs medical approaches to its diagnosis and treatment [4]. Consequently, researchers often rely on sparse and isolated case reports, requiring meticulous examination of available data to identify meaningful connections and patterns. We have established the known evidence of PWS with catatonia in the introduction and presented our case. What remains is to situate our findings alongside this literature for future reference.

The first case [8], a 17-year-old adolescent, presented with psychotic features (delusions of grandeur and visual hallucinations) that rapidly progressed to full catatonic stupor in one day. His catatonia changed features after the initiation of treatment with lorazepam, from stupor to motor excitability, as well as the persistence of positive psychosis. He had returned to baseline two weeks after treatment with lorazepam and risperidone. The second case [9], published in 2015, went through three catatonic episodes in her lifetime. At ages 15 and 16, both individuals experienced catatonic episodes stemming from depressive episodes, which were treated with lorazepam and haloperidol. Her third episode came following a manic episode. She was treated initially with lorazepam, but due to the lack of improvement in 10 days, the treatment was changed to eight courses of electroconvulsive therapy with lorazepam. She was at 70% of her baseline after finishing treatment and back to baseline six months later.

Our patient’s age corresponds with the onset of catatonia in the other two cases [8,9]. Notably, there were no risk factors before the catatonia, no infections, and no recent acute changes in her living situation. Additionally, the absence of psychotic features prior to the catatonic state is significant, given that her psychotic features resolved completely with the resolution of the catatonia, which contrasts with the presentations of both previous cases.

Another distinction lies in the nature of her catatonic episode. Unlike the stupor presentation described in the cases by Dhossche and Bouman [8] and Poser and Trutia [9], her catatonia manifested in a hyperactive state. In addition, her symptoms progressed gradually over several days, in contrast to the rapid onset within a single day observed in earlier reports.

Finally, the positive effect of benzodiazepines in catatonia [10,11] can explain the initial short response to midazolam [12]; her response to lorazepam matches what is known about catatonia treatment in the literature [10,13], and the return to her baseline within two weeks shows a positive outcome.

## Conclusions

Catatonia in children is both underrecognized and poorly understood, emphasizing the need for further research to understand its etiological causes. This case adds valuable insight to the growing body of literature on catatonia, particularly in relation to PWS. It also highlights the critical importance of comprehensively evaluating children displaying unusual behaviors to identify any underlying organic or neurological conditions rather than simply addressing the outward signs of agitation. However, further studies are required to provide a systematic analysis of the available data.
